# Surface In Situ Growth of Two-Dimensional/Three-Dimensional Heterojunction Perovskite Film for Achieving High-Performance Flexible Perovskite Solar Cells

**DOI:** 10.3390/nano15110798

**Published:** 2025-05-26

**Authors:** Zhiyu Zhang, Huijing Liu, Jing Liu, Jia Xu, Zhan’ao Tan, Jianxi Yao

**Affiliations:** 1 State Key Laboratory of Alternate Electrical Power System with Renewable Energy Sources, North China Electric Power University, Beijing 102206, China; 120222211096@ncepu.edu.cn (Z.Z.); 1182111006@ncepu.edu.cn (H.L.); liujing38@foton.com.cn (J.L.); xujia@ncepu.edu.cn (J.X.); 2 Beijing Advanced Innovation Center for Soft Matter Science and Engineering, Beijing University of Chemical Technology, North Third Ring Road 15, Beijing 100090, China

**Keywords:** flexible perovskite solar cells, 2D/3D heterojunction perovskite, passivate defects, stability

## Abstract

Organic–inorganic hybrid flexible perovskite solar cells (F-PSCs) have garnered considerable interest owing to their exceptional power conversion efficiency (PCE) and stable operational characteristics. However, F-PSCs continue to exhibit significantly lower PCE than their rigid counterparts. Herein, we employed 3-chloro-4-methoxybenzylamine hydrochloride (CMBACl) treatment to grow in situ two-dimensional (2D) perovskite layers on three-dimensional (3D) perovskite films. Through comprehensive physicochemical characterization, including X-ray diffraction (XRD), X-ray photoelectron spectroscopy (XPS), and photoluminescence (PL) mapping, we demonstrated that CMBACl treatment enabled the in situ growth of two-dimensional (2D) perovskite layers on three-dimensional (3D) perovskite films via chemical interactions between CMBA^+^ cations and undercoordinated Pb^2+^ sites. The organic cation (CMBA^+^) bound to uncoordinated Pb^2+^ ions and residual PbI_2_, while the chlorine anion (Cl^−^) filled iodine vacancies in the perovskite lattice, thereby forming a high-quality 2D/3D heterojunction structure. The CMBACl treatment effectively passivated surface defects in the perovskite films, prolonged charge carrier lifetimes, and enhanced the operational stability of the photovoltaic devices. Additionally, the hybrid 2D/3D architecture also improved energy band matching, thereby boosting charge transfer performance. The optimized flexible devices demonstrated a PCE of 23.15%, while retaining over 82% of their initial efficiency after enduring 5000 bending cycles under a 5 mm curvature radius (R = 5 mm). The unpackaged devices retained 94% of their initial efficiency after 1000 h under ambient conditions with a relative humidity (RH) of 45 ± 5%. This strategy offers practical guidelines for selecting interface passivation materials to enhance the efficiency and stability of F-PSCs.

## 1. Introduction

Perovskite solar cells (PSCs) have emerged as a prominent focus in photovoltaic technology research owing to their exceptional power conversion efficiency (PCE) and cost-effectiveness [1,2,3,4]. The PCE of PSCs has significantly improved, rising from 3.8% to a certified record of 26.9% over the past decade [5,6], showing a substantial development potential and commercial application prospect. The inherent flexibility of perovskite materials and their compatibility with low-temperature solution processing offer distinct advantages for flexible perovskite solar cells (F-PSCs), with single-junction F-PSCs achieving a record PCE of 24.84% [7]. The roll-to-roll manufacturability of F-PSCs enables scalable production at reduced costs, positioning them as promising candidates for applications in building-integrated photovoltaics, aerospace, and related fields [8]. Due to the evaporation of halide ions and organic cations, the perovskite-annealing process will induces defects, especially surface defects, resulting in non-radiative recombination [9,10,11,12,13]. These surface defects further accelerate moisture ingress into the perovskite layer under ambient conditions, degrading both efficiency and operational stability [14,15]. Furthermore, the defects rapidly induce irreversible lattice damage in the perovskite film during bending, which further decreases the mechanical durability of flexible devices [16,17]. Additionally, the low thermal tolerance of flexible substrates limits perovskite’s crystallinity compared with rigid substrates processed under identical conditions [18,19,20]. Consequently, F-PSCs exhibit a substantially lower PCE than rigid devices, underscoring the urgent need for strategies to improve their efficiency and stability.

In recent years, numerous approaches have been proposed to enhance the performance and durability of F-PSCs, including substrate optimization [21,22], additive engineering [20,23,24], and defect passivation. Among these methods, surface defect passivation has emerged as a critical factor in PSC research [25,26]. In particular, the in situ growth of two-dimensional (2D) perovskite layers on three-dimensional (3D) perovskite films has emerged as a viable interface modification strategy [26,27]. For 2D perovskite structures, hydrophobic organic amine cations (e.g., BA^+^ [28] and PEA^+^ [29]) replace monovalent cations, enhancing moisture resistance and phase stability [26,30]. In addition, the 2D/3D heterojunction further optimizes the energy level alignment and charge transport kinetics [31,32]. Therefore, suitable large-sized organic cations can be chosen to interact with excess PbI_2_ in the 3D perovskite film, forming a 2D perovskite layer that enhances the stability and efficiency of the devices. Shi et al. deposited 3-chlorophenyliodide ammonium (3-CBAI) on a 3D perovskite film to grow a low-dimensional perovskite capping layer in situ, enhancing the environmental and mechanical stability, and achieving a PCE increase from 18.4% to 21.0% [33]. Ge et al. engineered a ferroelectric two-dimensional perovskite architecture through pyridine heterocyclic coordination with residual PbI_2_, thereby enhancing the built-in electric field within the perovskite film, leading to a 23.01% PCE for F-PSCs with 2D/3D heterojunctions [34]. Despite the enhanced efficiency in F-PSCs, surface defects persist as critical bottlenecks limiting the efficiency of F-PSCs.

In this study, we used organic ammonium salt 3-chloro-4-methoxybenzylamine hydrochloride (CMBACl) to react with excess PbI_2_ on the perovskite film surface, creating a 2D perovskite layer. The organic cation (CMBA^+^) bound to uncoordinated Pb²^+^ ions, while the chlorine anion (Cl^−^) filled iodine vacancies in the perovskite lattice, thereby forming a high-quality 2D/3D heterojunction structure, a chlorine- and methoxy-functionalized ammonium salt enabling simultaneous defect passivation, 2D/3D heterojunction engineering, and humidity resistance in F-PSCs. Moreover, the 2D interlayers improved the energy level alignment between Spiro-OMeTAD and perovskite, boosted charge extraction, and prevented moisture ingress. Consequently, the optimized F-PSCs achieved the highest PCE of 23.15%. These devices demonstrated remarkable durability, maintaining over 82% of their original efficiency even after undergoing 5000 bending cycles at R = 5 mm. When subjected to 1000 h of testing at 45 ± 5% relative humidity, the modified F-PSC retained more than 94% of its initial PCE, while the unmodified device exhibited 78.9% efficiency retention under identical conditions.

## 2. Materials and Methods

### 2.1. Materials

Lead (II) bromide (PbBr_2_; 99%), cesium iodide (CsI; 99.0%), lead (II) iodide (PbI_2_; 99.99%), and 3-chloro-4-methoxybenzylamine hydrochloride (CMBACl; 97%) were purchased from TCI (Shanghai, China). The chlorobenzene (99.8%) and isopropanol (IPA; 99.5%), dimethyl sulfoxide (DMSO; 99.8%), and N, N-dimethylformamide (DMF; 99.8%) were sourced from Alfa Aesar (Shanghai, China). The 2-(2-aminothiazol-4-yl) acetic acid hydrochloride (ATACl; 98%) was acquired from Macklin (Shanghai). Additionally, bis(trifluoromethane)sulfonimide lithium salt (Li-TFSI), Tris(2-(1H-pyrazol-1-yl)-4-tert-butylpyridine)-cobalt(III)Tris(bis-(trifluoromethylsulfonyl)imide) (FK 209 Co(III) TFSI salt), and formamidinium iodide (FAI; 99.5%) were sourced from Xi’an Polymer Light Technology Corporation (Xi’an, China). Other chemical reagents included methylamine chloride (MACl; 99.5%) and methylammonium bromine (MABr; 99.5%) from Advanced Election Technology Co., Ltd. (Shenzhen, China), along with spiro-OMeTAD (99.86%) supplied by the same vendor. 4-tert-butylpyridine (tBP) was procured from Borun (Shenzhen, China), with the remaining components obtained from Sigma-Aldrich or Sinopharm Chemical Reagent (Shanghai, China). All compounds were employed as-received without additional purification.

### 2.2. Solution Preparation

Aqueous SnO_2_ colloids were formulated through volumetric dilution (1:3 *v*/*v*) of the precursor dispersion with deionized water, followed by the controlled addition of ATACl at gradient concentrations. The precursor solution of (FAPbI_3_)_0.93_(MAPbBr_3_)_0.05_(CsPbI_3_)_0.02_ was prepared by dissolving stoichiometric amounts of CsI, FAI, PbI_2_, PbBr_2_, MABr, and MACl in a 4:1 (*v*/*v*) DMF/DMSO solvent mixture. The CMBACl solutions with concentration gradients (1–3 mg/mL) were formulated via IPA dissolution under an inert atmosphere after 1 h of nitrogen-purged agitation. The Spiro-OMeTAD solution was formulated by dissolving 73.5 mg of the Spiro-OMeTAD powder in 1 mL of chlorobenzene, with the sequential addition of 29 μL of 4-tert-butylpyridine, 17 μL of Li-TFSI (520 mg/mL in acetonitrile), and 8 μL of the Co (III)-FK102 complex (400 mg/mL in acetonitrile).

### 2.3. Device Fabrication

The PET/ITO flexible substrate (23 mm × 23 mm) was washed successively with deionized water and anhydrous ethanol for 30 min. The treated PET/ITO flexible substrates were purged with dry air, followed by a 45 min UV–ozone treatment for surface cleaning. A 150 μL SnO_2_ colloidal dispersion was deposited via spin coating onto the PET/ITO flexible substrate, followed by thermal annealing at 150 °C for 30 min to form the electron transport layer. Subsequently, the perovskite active layer was fabricated through a two-step spin-coating protocol (1000 rpm initial acceleration) using stoichiometric precursor solutions for 10 s and followed by 4500 rpm for 30 s. During terminal 15 s of the spin-coating process, 150 μL of chlorobenzene was dropped onto the functionalized substrates. Post-deposition thermal annealing at 120 °C for 20 min preceded the spin coating of the concentration gradient CMBACl solutions (3500 rpm; 30 s) onto the perovskite active layers. The hole transport layer (HTL) was formed by spin coating the Spiro-OMeTAD solution at 3500 rpm for 25 s onto the perovskite films, followed by the vacuum deposition of 80 nm of Au electrodes through thermal evaporation. Flexible photovoltaic devices with 0.09 cm² active areas were thereby fabricated. All steps were performed in an air glovebox maintained at 25 °C and RH 10%. The characterization information is in the Supplementary Material.

## 3. Results and Discussion

**Figure 1:**
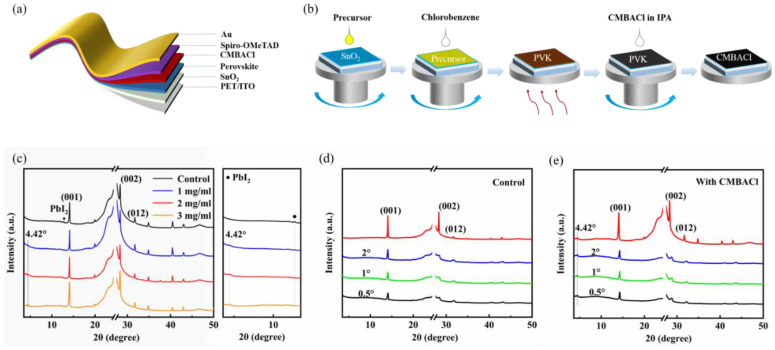
(**a**) Structural schematic of F-PSC. (**b**) Schematic illustration of post-treatment for perovskite film. (**c**) XRD patterns of control and CMBACl-treated perovskite films. GIXRD patterns of (**d**) control and (**e**) CMBACl-treated perovskite films at different incident angles.

Figure 1a illustrates the n-i-p architecture of the F-PSCs: PET/ITO/SnO_2_/3D perovskite/2D perovskite/Spiro-OMeTAD/Au. The fabrication process is shown in Figure 1b. The chemical structure of CMBACl is shown in Figure S1. Post-treatment involved spin coating CMBACl isopropanol solutions at varying concentrations (1, 2, and 3 mg/mL) onto the perovskite layer. The cross-sectional SEM image of the rigid device in Figure S2 reveals a perovskite absorber layer thickness of 740 nm. The effects of CMBACL were analyzed by comparing the untreated control with the CMBACL-treated perovskite films.

Figure 1c presents the XRD patterns of the untreated and CMBACl-treated perovskite films to assess the post-treatment effects. The XRD patterns of both the pristine and CMBACl-treated perovskite films exhibited characteristic α-FAPbI_3_ (001)/(002)/(012) reflections at 13.96°, 28.14°, and 31.9° (2θ), confirming a well-crystallized perovskite film. The consistency of the perovskite peak intensity suggested that the CMBACl post-treatment minimally affected the bulk crystalline of the perovskite films. Additionally, in the magnification XRD patterns, a faint diffraction peak for PbI_2_ was detected at 12.7° in the control perovskite film samples. Following the CMBACl treatment, the PbI_2_ peak intensity decreased significantly, while a new (010) diffraction peak emerged at 4.42°, characteristic of 2D perovskite formation [35]. We investigated the interaction between CMBACL molecules and PbI_2_ by preparing (CMBACl)_2_-PbI_2_ complex films. We observe that the diffraction peaks at 4.42°, 8.79°, and 13.21° were detected in the XRD spectra in Figure S3, confirming the formation of a 2D perovskite (CMBAC_2_PbI_2_Cl_2_). By analyzing the XRD results, we deduced the following reaction equations:

2CMBACl + PbI_2_ → CMBA_2_PbI_2_Cl_2_ (1)

When a 3D perovskite film was treated by CMBACl, an in situ reaction occurred between CMBACl and excess PbI_2_, as shown in Equation (1), forming 2D perovskite CMBA_2_PbI_2_Cl_2_. Surface crystallographic examination of the perovskite films was performed before and after the 2 mg/mL CMBACl treatment via grazing-incidence X-ray diffraction (GIXRD) (Figure 1d,e). The crystal structure and orientation of the perovskite film surface remained unchanged after the CMBACl treatment, indicating that CMBACl merely interacted with the excess PbI_2_ in the perovskite film to form 2D perovskite. The X-ray diffraction results indicate that the in situ growth of the 2D perovskite, achieved through the CMBACl treatment, effectively modulated the surface of the 3D perovskite, which played an active role in improving the crystallinity of the perovskite film.

**Figure 2:**
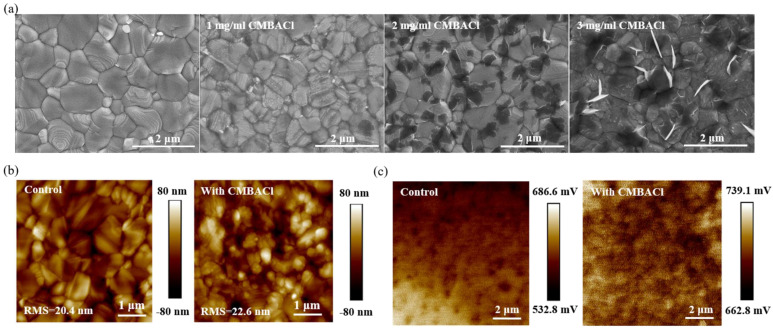
(**a**) SEM images of control, 1 mg/mL, 2 mg/mL, and 3 mg/mL CMBACl-treated perovskite films. (**b**) AFM and (**c**) KPFM images of control and CMBACl-treated perovskite films.

The scanning electron microscopy (SEM) images in Figure 2a present the effect of CMBACl with different concentrations on the surface morphology of the perovskite films. The untreated control film exhibited a pinhole-free, compact surface with sporadic white lamellar grains at the grain boundaries, attributed to excess PbI_2_ [36]. In comparison, the CMBACl-treated perovskite film showed similar compact textures to the control film, and some black layered grains were observed, and a gradual accumulation of black layered grains was observed as the CMBACl concentration increased. Combined with the XRD results, the black layered grains indicated the formation of 2D perovskite. Atomic force microscopy (AFM) images of the perovskite films were also obtained to test the surface roughness, as illustrated in Figure 2b. The surface roughness of the perovskite film slightly increased from 20.4 nm to 22.6 nm through the CMBACl treatment, attributed to the formation of 2D perovskite layers. Kelvin probe force microscopy (KPFM) measurement was carried out to explore the film surface potential. Figure 2c illustrates the perovskite film’s increased surface potential, implying a diminished work function, which promoted efficient interfacial charge transport [37].

**Figure 3:**
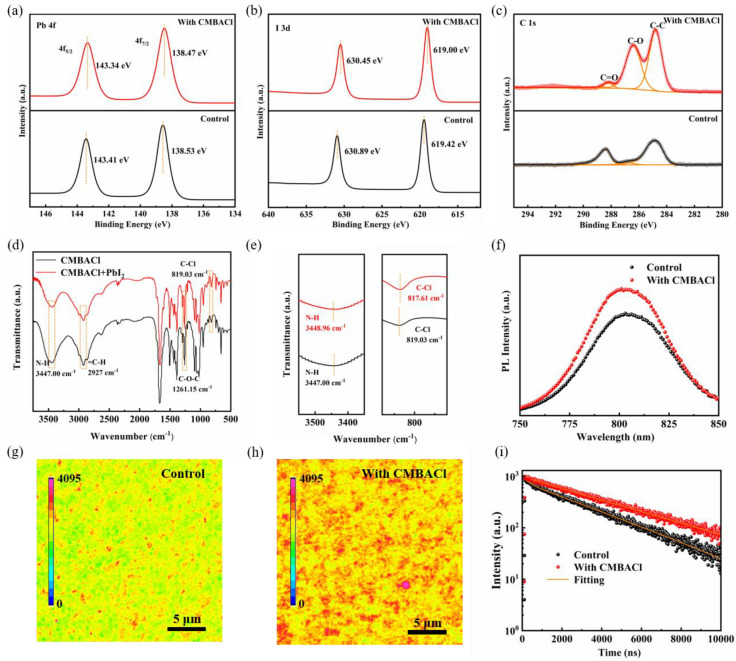
XPS spectra of (**a**) Pb 4f, (**b**) I 3d, and (**c**) C1s for control and CMBACl-treated perovskite films. (**d**) FTIR spectra and (**e**) magnified FTIR spectra of PbI_2_ and (CMBACl)_2_-PbI_2_ (CMBACl and PbI_2_ dissolved in DMF/DMSO mixed solution). (**f**) Steady-state PL of control and CMBACl-treated perovskite films. PL mappings of (**g**) control and (**h**) CMBACl-treated perovskite films. (**i**) TRPL decay curves of control and CMBACl-treated perovskite films.

X-ray photoelectron spectroscopy (XPS) analysis was conducted to explore the chemical interaction between CMBACl and PbI_2_ within the perovskite layer. Figure 3a,b show the Pb 4f and I 3d spectra of the control and CMBACl perovskite layers. For the control film, the binding energy peaks of Pb 4f_5/2_ and Pb 4f_7/2_ were located at 138.53 and 143.41 eV, and the I 3d binding energy peaks were located at 619.42 eV and 630.89 eV, respectively. Compared with the unmodified perovskite film, the binding energy peaks of Pb 4f and I 3d in the CMBACl-treated perovskite shifted marginally toward a lower binding energy, thereby confirming the interplay between CMBACl and PbI_2_. As shown in Figure 3c, an additional C-O binding energy peak after the CMBACl treatment indicated the presence of CMBA^+^ on the perovskite surface. Notably, we detected that the C=O binding energy peak originating from oxygen and moisture in the modified perovskite film was considerably suppressed [12], indirectly demonstrating that the CMBACl treatment could enhance the hydrophobicity and reduce the degradation of the perovskite film. As shown in Figure S4, the spectrum of the control perovskite film showed a prominent binding energy peak of Cl 2p. Two new peaks were detected after the CMBACl treatment, which confirmed the existence of CMBACl. As illustrated in Figure S5, the water contact angle of the perovskite film following the CMBACl treatment increased from 62.8° to 72.9°. This result indicates that the CMBACl-treated method increased the perovskite film’s moisture resistance ability.

Fourier-transform infrared (FTIR) spectra were obtained to identify the interaction between CMBACl and PbI_2_. Figure 3d shows the FTIR spectra of the CMBACl, PbI_2_, and (CMBACl)_2_-PbI_2_ samples. After PbI_2_ was mixed with CMBACl, the stretching vibration peak (3447.00 cm^−1^) belonging to the N-H functional groups of CMBACl shifted toward lower wavenumbers (3448.96 cm^−1^). Furthermore, the C-Cl functional group was located at 819.03 cm^−1^ in the control sample, which shifted to 817.61 cm^−1^ in the (CMBACl)_2_-PbI_2_ samples. These results further proved the interaction between the CMBACl and PbI_2_. Stable photoluminescence (PL) and PL mapping prepared on a glass substrate were performed to analyze the charge carrier dynamics in the 2D/3D heterostructure. The enhanced PL intensity in the CMBACl-treated perovskite films (Figure 3f) originated from dual defect–passivation mechanisms: organic ammonium groups coordinated with under-bonded Pb²^+^, while Cl^−^ filled iodine vacancies on the 3D perovskite surface. The situ-formed 2D/3D heterojunction simultaneously suppressed non-radiative recombination pathways and optimized interfacial energy alignment, enabling more efficient radiative recombination through improved charge confinement and surface passivation [38]. In Figure S6, the concentrations of the CMBACl solutions increased to 4 mg/mL, and two emission peaks at around 510 nm and 565 nm belonging to 2D perovskite were detected, further verifying the formation of 2D perovskite on the 3D perovskite film. PL mapping was conducted to assess the effect on the perovskite films of the CMBACl treatment. In Figure 3g,h, the CMBACl-treated perovskite films show a more uniform and higher emission intensity in the selected region compared with the control film. The results exhibit that the CMBACl treatment could passivate defects and enhance carrier transport. Furthermore, we tested the time-resolved photoluminescence (TRPL) to further investigate the carrier lifetime, as depicted in Figure 3i, and the fitted parameters are summarized in Table S1. The TRPL decay curves were fitted by the biexponential model (2):

(2) $$I(t)=A_{1}{exp}^{\left(\frac{-t}{\tau_{1}}\right)}+A_{2}{exp}^{\left(\frac{-t}{\tau_{2}}\right)}$$

where A_1_ and A_2_ stand for the relative amplitude, and τ_1_ and τ_2_ are the fast and slow attenuation components, related to non-radiation recombination and radiation recombination, respectively. The average carrier lifetime (τ_ave_) can be obtained from Equation (3):

(3) $$\tau_{ave}=\frac{\left(A_{1}{\tau_{1}}^{2}+A_{2}{\tau_{2}}^{2}\right)}{\left(A_{1}\tau_{1}+A_{2}\tau_{2}\right)}$$

The τ_ave_ of the CMBACl-treated perovskite film (3892.16 ns) was significantly higher than that of the control sample (2587.92 ns). Therefore, the in situ growth of 2D perovskite layers on the 3D perovskite films through the ammonium salt post-treatment was demonstrated as a valid strategy for diminishing the trap-state density and prolonging the carrier lifetime, leading to an elevated open-circuit voltage (*V_OC_*).

**Figure 4:**
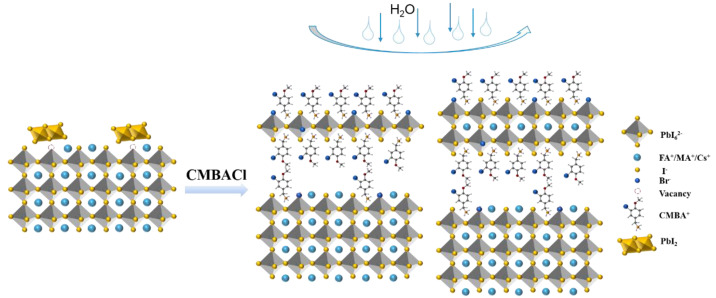
Schematic of reaction mechanism for CMBACl post-treatment forming 2D/3D heterojunction perovskite film.

According to the results discussed above, the detailed mechanism of the interaction between CMBACl and the perovskite films is illustrated in Figure 4. Many surface defects and residual PbI_2_ existed on the control perovskite films, leading to serious non-radiative recombination. During the treatment processes, CMBACl dissociated into CMBA^+^ and Cl^−^ in IPA, reacting with the excess PbI_2_ to form a 2D perovskite layer on the 3D perovskite surface via in situ growth. Meanwhile, CMBA^+^ also bound to the uncoordinated Pb^2+^, while Cl^−^ in the CMBACl filled the iodine vacancies in the perovskite lattice, thus enabling surface defects to be passivated, thereby reducing non-radiative recombination losses and enhancing charge carrier mobility. In addition, the 2D perovskite phase further improved the moisture resistance and phase stability compared with the pure 3D films, enhancing the overall device durability. In summary, this multifunctional ammonium salt strategy achieved dual benefits: defect elimination and 2D/3D heterojunction formation, collectively improving the device’s longevity.

**Figure 5:**
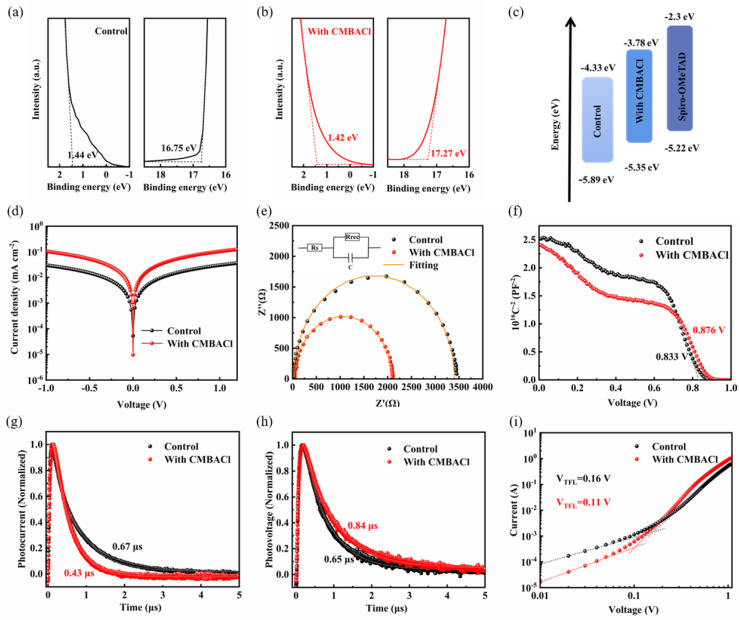
UPS spectra of (**a**) control and (**b**) CMBACl-treated perovskite films. (**c**) Energy level scheme for control and CMBACl-treated films extracted from UPS data. (**d**) Dark *J-V*, (**e**) EIS, (**f**) Mott–Schottky, (**g**) TPC, and (**h**) TPV curves for control and CMBACl-treated F-PSCs. (**i**) SCLC curves of hole-only devices based on control and CMBACl-treated F-PSCs.

Ultraviolet photoelectron spectroscopy (UPS) was employed to probe the energy level alignment of the 2D/3D heterojunction, a key factor governing charge transport in F-PSCs. As shown in the ultraviolet–visible (UV–vis) absorption spectra (Figure S7), the absorption intensity of the films experienced a negligible increase. The band gap values of the control and CMBACl-treated perovskite films were calculated as 1.56 eV and 1.57 eV, respectively, obtained from Tauc plots (Figure S8), confirming their preserved α-phase crystallinity. In the UPS spectra (Figure 5a,b), CMBACl adjusted the perovskite film’s work function, elevating it from −4.47 to −3.95 eV; this aligns with the surface potential increases detected via KPFM. The 2D/3D heterostructure obtained a valence band maximum (VBM) of −5.35 eV and a conduction band minimum (CBM) of −3.78 eV, with the energy level alignment mapped in Figure 5c. This interfacial engineering reduced the energy offset between the pristine perovskite and hole transport layers by 0.42 eV, thereby boosting the photogenerated carrier extraction efficiency and elevating the *V*_OC_ through improved energy level matching.

Optoelectronic characterization of the F-PSCs was systematically performed to prove the effect on the enhanced performance of the devices with the CMBACl treatment. As revealed in Figure 5d, a decreased dark current density was observed in the CMBACl-modified devices compared with the untreated devices through dark *J-V* analysis, suggesting mitigated leakage currents and suppressed non-radiative recombination [39]. As shown in Figure 5e, Nyquist plots were generated by electrochemical impedance spectroscopy (EIS) to explore the charge transfer and recombination. Table S2 summarizes the corresponding parameters. The device with the CMBACl treatment showed a smaller charge transfer resistance (R_CT_), mainly caused by the reduced defect densities, reflecting its improved charge transfer ability. As presented in Figure 5f, the Mott–Schottky plot analysis was employed to obtain the built-in voltage (*V_bi_*) of the devices. The *V_bi_* of the CMBACl-treated device was enhanced to 0.876 V. A higher *V_bi_* indicated a stronger built-in electric field between the HTL and perovskite layer, facilitating the charge extraction efficiency, which was consistent with the improved *V*_OC_ [40]. In addition, transient photocurrent (TPC) and transient photovoltage (TPV) measurements were performed to assess the dynamics of the charge carriers within the F-PSCs. The TPC decay lifetimes decreased from 0.67 μs (control) to 0.43 μs (CMBACl-treated F-PSC) (Figure 5g), indicating accelerated charge extraction. The TPV of the CMBACl-treated device was enhanced to 0.84 μs, demonstrating suppressed non-radiative recombination through CMBACl passivation, as shown in Figure 5h. Space-charge-limited current (SCLC) measurement on the structure of PET/ITO/PEDOT:PSS/Perovskite/Spiro-OMeTAD/Au was carried out to analyze the trap-fitted limit voltages (*V_TFL_*) and trap density (*N_t_*) in the perovskites following Equation (4) [41]:

(4) $$N_{t}=\frac{2{\varepsilon \varepsilon }_{0}V_{TFL}}{eL^{2}}$$

where ε and ε_0_ represent the vacuum permittivity and dielectric constant, respectively, *e* signifies the elementary charge, and *L* represents the thickness of the absorber layer. As depicted in Figure 5i and Table S3, the *V_TFL_* of the perovskite films treated by CMBACl (1.10 V) was conspicuously lower than that of the control film (1.14 V). The N_t_ value in the perovskite film dramatically decreased from 1.1 × 10^15^ cm^−3^ to 6.8 × 10^14^ cm^−3^ after the CMBACl treatment, indicating CMBACl’s passivation action.

**Figure 6:**
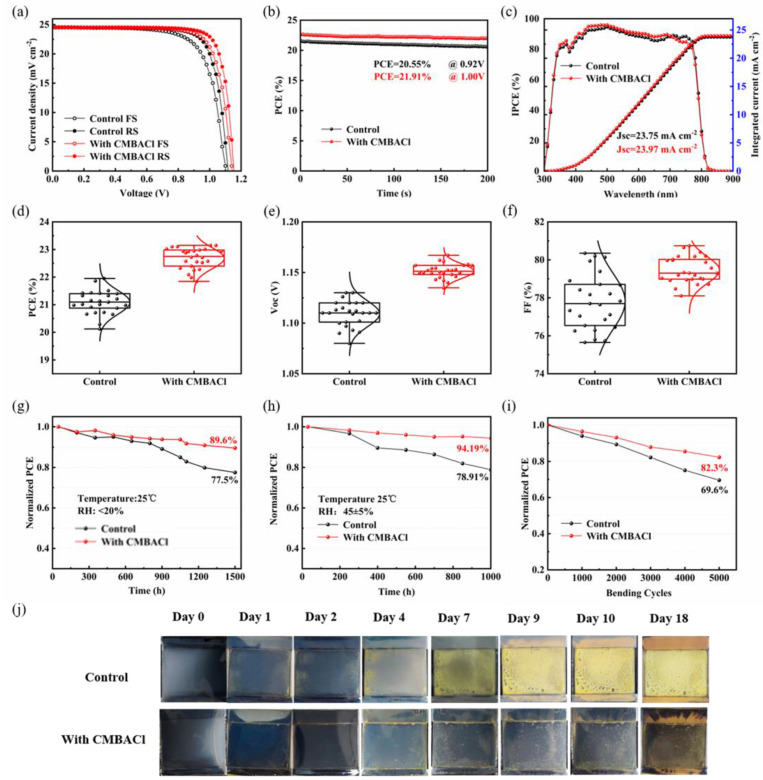
(**a**) *J-V* curves in reverse and forward scan directions, (**b**) stable output curves, and (**c**) IPCE and integrated current density spectra of control and CMBACl-treated F-PSCs. Statistical (**d**) PCE, (**e**) *V*_OC_, and (**f**) *FF* plots of F-PSCs control and CMBACl-treated F-PSCs. Long-term stability of the F-PSCs (based on a single device). (**g**) RH < 20% and (**h**) RH = 45 ± 5% in ambient environment. (**i**) Bending stability of F-PSCs (R = 5 mm, in N_2_ atmosphere at 25 °C). (**j**) Photographs of control and CMBACl-treated perovskite films aged in ambient environment with 80% RH.

Figure 6a shows the *J-V* curves of the champion and control devices (0.09 cm^−2^ active areas; AM 1.5G), and the corresponding parameters are summarized in Table S4. The PCE of the control device was 21.86%, whereas the CMBACl-treated device exhibited an excellent photovoltaic performance, with a *V*_OC_ of 1.16 V, a short-circuit current density (*J*_sc_) of 24.92 mA/cm^2^, an *FF* of 80.21%, and a PCE of 23.15%. These performance improvements were attributed to the strong passivation effects of CMBACl. The hysteresis index (HI) of the F-PSCs decreased from 7.5% (control) to 6.2% (CMBACl-treated F-PSC). The corresponding stabilized output current and efficiency at the maximum power point are presented in Figure 6b. Compared with the control device, the optimized device produced a PCE of 21.91% after a duration of 300 s under the maximum power point of 1 V, demonstrating good operation stability. As shown in Figure 6c, the integrated current densities obtained from the incident photon-to-electron conversion efficiency (IPCE) spectra for the control and CMBACl-treated devices were 23.75 mA cm^−2^ and 23.97 mA/cm^2^, respectively, which agreed with the *J-V* measurements. Statistical analysis of 30 devices (Figure 6d–f) confirmed the enhanced reproducibility of the CMBACl-treated F-PSCs, with consistently higher PCE, *V*_OC_, and *FF* values compared with the control F-PSCs. The enhanced device performance and improved reproducibility in the CMBACl-modified systems stemmed from three key factors: interfacial defect passivation, enhanced charge transport efficiency, and significantly suppressed non-radiative recombination pathways [42,43].

Long-term operational stability and flexibility are critical for advancing F-PSCs toward commercialization. Figure 6g demonstrates the operational stability of the CMBACl-modified devices, which retained 89.6% of their initial PCE after 1500 h at 20% RH, significantly outperforming the control devices (77.5% PCE retention). Under conditions of approximately 45 ± 5% RH at room temperature, as shown in Figure 6h, the F-PSCs treated with CMBACl maintained 94.1% of their initial efficiency after 1000 h, while the control device remained at about 78.9%. The mechanical flexibility of the flexible devices was evaluated through cyclic bending tests (R = 5 mm, in N_2_), with their curvature retention exceeding 95% after 10,000 cycles. Figure 6i shows that the CMBACl-treated and control devices retained 82.3% and 69.9% of their initial PCE after 5000 bending cycles, respectively. The F-PSCs with the CMBACl treatment showed excellent mechanical durability, which benefited from the decreased trap density and enhanced transport charge. Furthermore, under conditions of 80 ± 2% RH and room temperature, the unpackaged perovskite films with the CMBACl treatment showed better humidity stability, as illustrated in Figure 6j. The CMBACl-treated film remained black after 18 days, in contrast with the control film, which turned fully yellow after 10 days. The above results indicate that the CMBACl treatment could protect the perovskite film from destruction by moisture to improve device stability. Future efforts should focus on the scalable fabrication of 2D/3D heterostructures via roll-to-roll processes while exploring lead-free alternatives for eco-friendly applications. Characterization under combined stressors (humidity, bending, and UV) will deepen our mechanistic understanding.

## 4. Conclusions

In conclusion, a surface modification method was employed to fabricate 2D/3D perovskite heterostructures, enhancing the performance of the F-PSCs. CMBACl-based surface optimization significantly enhanced the device performance through synergistic defect passivation and interfacial modulation. CMBACl serves as a multifunctional passivation agent, reacting with excess PbI_2_ to form a 2D/3D heterostructure that simultaneously enhances charge transport kinetics, suppresses non-radiative recombination, and reduces the trap state density. The optimized F-PSCs obtained a champion PCE of 23.15%, with a high *V*_OC_ of 1.16 V. The treated devices demonstrated exceptional operational stability (94.1% PCE retention after 1000 h at 45 ± 5% RH) and mechanical durability (82% efficiency retention after 5000 bending cycles at R = 5 mm). Interfacial engineering was demonstrated to play a decisive role in perovskite photovoltaics, wherein the developed scalable passivation strategy enabled high-performance flexible solar cells to progress toward commercial viability.

## Data Availability

The data that support the findings of this study are available from the corresponding author upon reasonable request.

## Supplementary Materials

The following supporting information can be downloaded at: https://www.mdpi.com/article/10.3390/nano15110798/s1. Figure S1: Molecular structures of CMBACl; Figure S2: The cross-sectional SEM of the device on the rigid substrate; Figure S3: XRD patterns of CMBACl and (CMBACl)_2_-PbI_2_ (mole ratio of CMBACl:PbI_2_ = 2:1) films; Figure S4: XPS spectra of Cl 2p for control and CMBACl perovskite films; Figure S5: Water contact angle of control and CMBACl perovskite films; Figure S6: PL spectra of control and CMBACl perovskite films; Figure S7: UV−vis absorption spectra of control and CMBACl perovskite films; Figure S8: Tauc spectra of control and CMBACl treated perovskite films; Figure S9: Actual F-PSCs images at 5 mm curvature radii; Table S1. Fitted parameters of TRPL curves; Table S2: Summary of EIS parameters based on Control and CMBACl treated devices; Table S3: Summary of SCLC parameters for Control and CMBACl treated devices; Table S4: Detailed photovoltaic parameters of F-PSCs based on Control and CMBACl treated in forward and reverse scans; Table S5: Comparison of Photovoltaic Performance of Recent 2D/3D Perovskite Solar Cells. See Refs. [44,45,46,47,48,49,50,51,52].
