# Supplementary material for: Surface In Situ Growth of Two-Dimensional/Three-Dimensional Heterojunction Perovskite Film for Achieving High-Performance Flexible Perovskite Solar Cells
Source: Nanomaterials (Basel). 2025 May 26;15(11):798. doi: 10.3390/nano15110798 (PMC12156928; doi:10.3390/nano15110798)
Supplement: Supplementary file 1 [file nanomaterials-15-00798-s001.zip › nanomaterials-3644406-supplementary.pdf]

Supporting Information

Device Characterization

X-ray diffraction (XRD) patterns of the perovskite films were collected using a Rigaku SmartLab diffractometer, employing Cu Kα (1.5418 Å) radiation. X-ray photoelectron spectroscopy (XPS) analyses were conducted utilizing a PHI-5000C instrument by employing a monochromatized Al source (hν = 1486.6 eV). AFM measurement was recorded using an Atomic Force Microscope (5500, Agilent Technologies). A field emission high resolution scanning electron microscopy (SU8010, Hitachi) was used to obtain the scanning electron microscopy (SEM) images. UV-Visible absorption (UV-vis) spectra were acquired utilizing a Shimadzu UV2450 ultraviolet-visible spectrophotometer. The current-voltage (I-V) and current density−voltage (*J-V*) characteristic curves were acquired using a Keithley 2400 digital source meter in conjunction with a sunlight simulator (XES-300T1) operating under AM 1.5G conditions at 100 mW cm^{-2}. The *J-V* tests were conducted with a scanning speed of 0.02 V/s, covering a range from -0.02 V to 1.50 V, on devices with an active area of 0.0627 cm^2. Electrochemical impedance spectroscopy (EIS) measurements were performed using a Zahner electrochemical workstation (Kronach, Germany) with a bias potential of 1.00 V in the dark, spanning a frequency range from 1 Hz to 10 Hz. Capacitance-voltage (*C-V*) measurements were executed utilizing the Zahner electrochemical workstation under dark conditions, with a fixed frequency of 1 kHz and obtained in the forward direction from -0.2 to 1.1 V. Transient photocurrent/photovoltage (TPC/TPV) decays were captured through an all-in-one characterization platform known as Paios (TranPVC-M, Oriental Spectra Technology). The incident photon-to-electron conversion efficiency (IPCE) was measured in ambient air using a QE-R measurement system (Enli Technology). Steady-state photoluminescence (PL) spectra were measured at room temperature using a steady-state lifetime spectrofluorometer (Fluorolog 322, Horiba) with an excitation wavelength of 465 nm and 350 nm. Time resolved photoluminescence (TRPL) spectra were collected using a transient state spectrophotometer (FLS1000, Edinburgh Instruments) at an excitation wavelength of 465 nm. For the PL and TRPL measurements, the films were all deposited on the glass substrates. PL mapping was performed with a fluorescence lifetime imaging microscopy (ARsiMP-LSM, Nikon-PICOQUANT-Coherent). Fourier transform infrared spectroscopy (FTIR) spectra were acquired with the fourier transform infrared spectrometer (Excalibur 3100, Varian). The water contact angles measurements were obtained by using a contact angle analyzer (OCA15EC, Dataphysics)

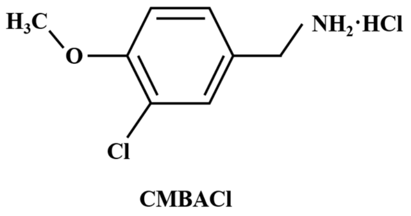

Figure S1. Molecular structures of CMBACl.

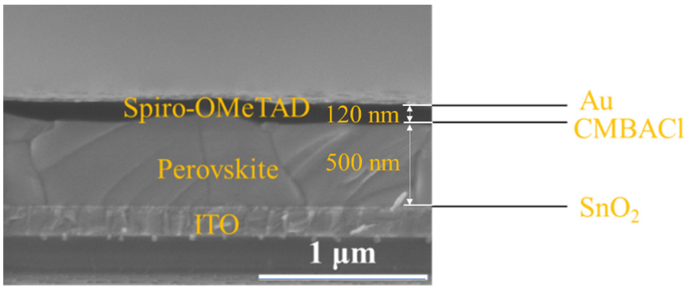

Figure S2. The cross-sectional SEM of the device on the rigid substrate.

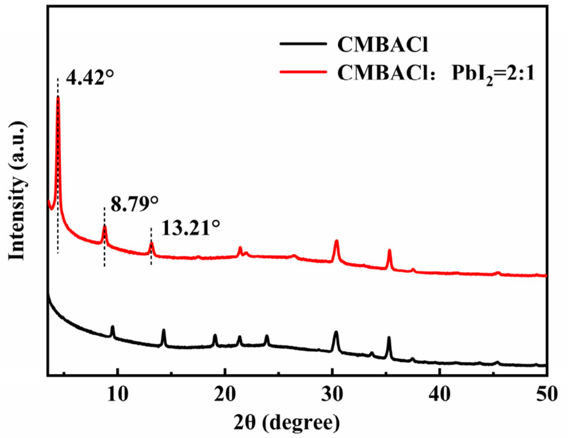

Figure S3. XRD patterns of CMBACl and $(CMBACl)_2$-PbI_2 (mole ratio of CMBACl:PbI_2=2:1) films.

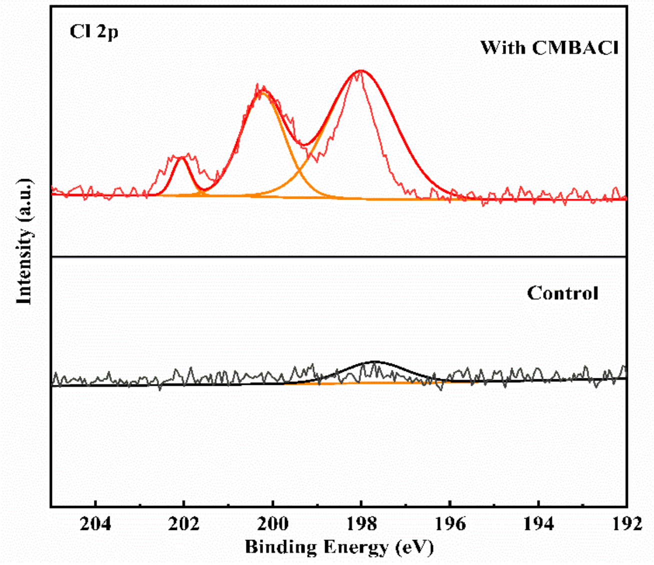

Figure S4. XPS spectra of Cl 2p for control and CMBACl perovskite films.

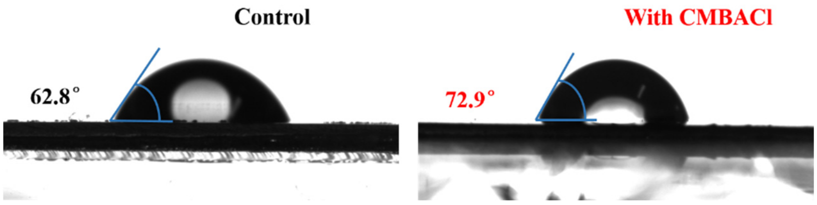

Figure S5. Water contact angle of control and CMBACl perovskite films.

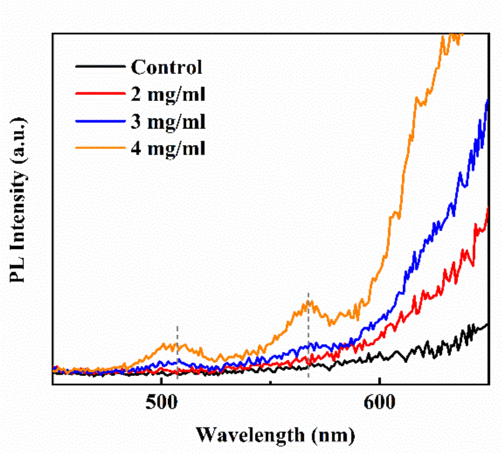

Figure S6. PL spectra of control and CMBACl perovskite films.

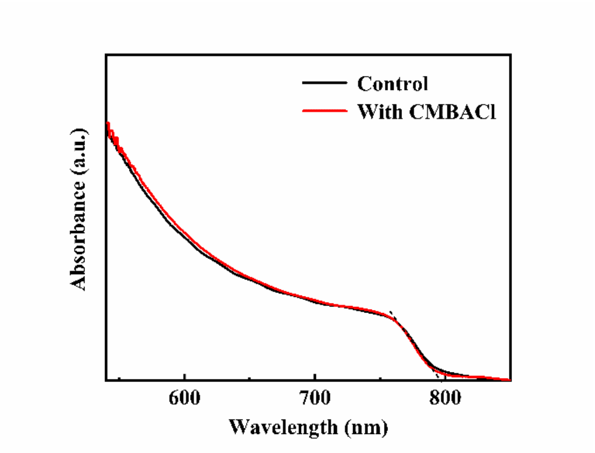

Figure S7. UV−vis absorption spectra of control and CMBACl perovskite films.

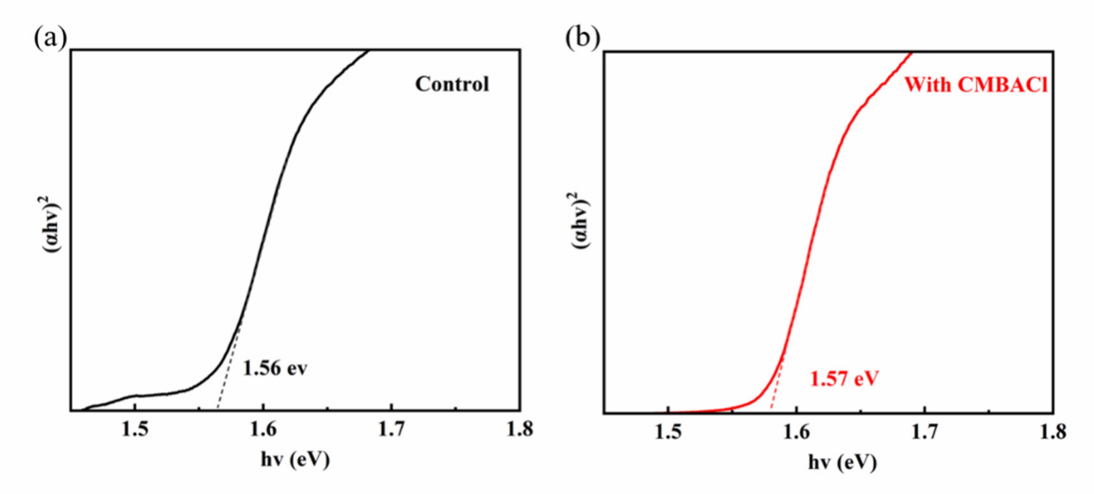

Figure S8. Tauc spectra of control and CMBACl treated perovskite films.

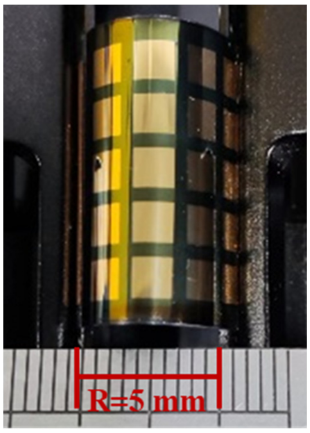

Figure S9. Actual F-PSCs images at 5 mm curvature radii.

Tables

Table S1. Fitted parameters of TRPL curves.

Sample	A_1	τ_1 (ns)	A_2	τ_2 (ns)	τ_{ave} (ns)
Control	147.74	304.71	749.99	3041.10	2587.92
2 mg/ml	26.19	1000.01	844.01	3981.21	3892.16

Table S2. Summary of EIS parameters based on Control and CMBACl treated devices.

Sample	R_{ct}（Ω）	R_S（Ω）	C（nF）
Control	6756	25.12	5.84
With CMBACl	3378	23.49	6.46

Table S3. Summary of SCLC parameters for Control and CMBACl treated devices.

Sample	V_{TFL} (V)	N_t (cm^{-3})
Control	0.10	1.09×10^{15}
With CMBACl	0.16	6.8×10^{14}

Table S4. Detailed photovoltaic parameters of F-PSCs based on Control and CMBACl treated in forward and reverse scans.

Devices		V_{OC} (V)	*FF* (%)	J_{SC} (mA cm^{-2})	PCE (%)	*HI*(%)
Control	FS	1.10	74.57	24.43	20.21	7.5
	RS	1.11	79.94	24.44	21.86	
With CMBACl	FS	1.14	75.97	24.99	21.71	6.2
	RS	1.16	80.21	24.92	23.15	

Table S5 Comparison of Photovoltaic Performance of Recent 2D/3D Perovskite Solar Cells

Reference	V_{oc} (V)	J_{sc} (mA/cm^2)	FF (%)	PCE (%)	2DPerovskite	Type	Publication Year
1	1.17	26.1	85.4	24.6	$(A6BfP)_8Pb_7I_{22}$	Rigid	2024
2	1.16	25.26	83.9	24.5	$DMePDAPbI_4$	Rigid	2023
3	1.18	25.70	81.8	24.9	$(4AP)PbI_4$	Rigid	2023
3	1.15	25.26	76.6	22.3	$(4AP)PbI_4$	Flexible	2023
4	1.16	25.87	86.2	25.9	$CHEA_2PbI_4$	Rigid	2024

5	1.07	19.95	73.0	16.1	$(PEI)_2[PbI_4]$	Rigid	2015
6	1.12	25.5	82.0	24.1	$BA_2FAPb_2I_7$/ $PA_2FAPb_2I_7$	Rigid	2024
7	1.18	25.71	83.2	25.3	-	Rigid	2024
8	1.20	25.09	82.0	24.3	-	Rigid	2022
9	1.20	24.34	84.0	24.5	$BA_2MA_2Pb_3I_{10}$	Rigid	2022
This work	1.16	24.92	80.2	23.2	$CMBA_2PbI_2Cl_2$	Flexible	2025

References

[1] Liu, C.; Yang, Y.; Chen, H.; Spanopoulos, I.; Bati, A.S.R.; Gilley, I.W.; Chen, J.; Maxwell, A.; Vishal, B.; Reynolds, R.P.; et al. Two-Dimensional Perovskitoids Enhance Stability in Perovskite Solar Cells. *Nature* **2024**, *633*, 359–364.

[2] Zhang, F.; Park, S.Y.; Yao, C.; Lu, H.; Dunfield, S.P.; Xiao, C.; Uličná, S.; Zhao, X.; Du Hill, L.; Chen, X.; et al. Metastable Dion-Jacobson 2D Structure Enables Efficient and Stable Perovskite Solar Cells. *Science* **2022**, *375*, 71–76.

[3] Yang, T.; Ma, C.; Cai, W.; Wang, S.; Wu, Y.; Feng, J.; Wu, N.; Li, H.; Huang, W.; Ding, Z.; et al. Amidino-Based Dion-Jacobson 2D Perovskite for Efficient and Stable 2D/3D Heterostructure Perovskite Solar Cells. *Joule* **2023**, *7*, 574–586.

[4] Wang, H.; Su, S.; Chen, Y.; Ren, M.; Wang, S.; Wang, Y.; Zhu, C.; Miao, Y.; Ouyang, C.; Zhao, Y. Impurity-Healing Interface Engineering for Efficient Perovskite Submodules. *Nature* **2024**, *634*, 1091–1095.

[5] Yao, K.; Wang, X.; Xu, Y.; Li, F. A General Fabrication Procedure for Efficient and Stable Planar Perovskite Solar Cells: Morphological and Interfacial Control by in-Situ-Generated Layered Perovskite. *Nano Energy* **2015**, *18*, 165–175.

[6] Sidhik, S.; Metcalf, I.; Li, W.; Kodalle, T.; Dolan, C.J.; Khalili, M.; Hou, J.; Mandani, F.; Torma, A.; Zhang, H.; et al. Two-Dimensional Perovskite Templates for Durable, Efficient Formamidinium Perovskite Solar Cells. *Science* **2024**, *384*, 1227–1235.

[7] Gong, C.; Chen, X.; Zeng, J.; Wang, H.; Li, H.; Qian, Q.; Zhang, C.; Zhuang, Q.; Yu, X.; Gong, S.; et al. Functional-Group-Induced Single Quantum Well Dion–Jacobson 2D Perovskite for Efficient and Stable Inverted Perovskite Solar Cells. *Advanced Materials* **2024**, *36*, 2307422.

[8] Azmi, R.; Ugur, E.; Seitkhan, A.; Aljamaan, F.; Subbiah, A.S.; Liu, J.; Harrison, G.T.; Nugraha, M.I.; Eswaran, M.K.; Babics, M.; et al. Damp Heat–Stable Perovskite Solar Cells with Tailored-Dimensionality 2D/3D Heterojunctions. *Science* **2022**, *376*, 73–77.

[9] Sidhik, S.; Wang, Y.; De Siena, M.; Asadpour, R.; Torma, A.J.; Terlier, T.; Ho, K.; Li, W.; Puthirath, A.B.; Shuai, X.; et al. Deterministic Fabrication of 3D/2D Perovskite Bilayer Stacks for Durable and Efficient Solar Cells. Science **2022**, 377, 1425–1430.
